# Identification of mixed anaerobic infections after inguinal hernia repair based on metagenomic next-generation sequencing: A case report

**DOI:** 10.1515/med-2023-0887

**Published:** 2024-01-12

**Authors:** Jun Zhang, Yuming Zhou, Zhenzhou Zhong, Yan Lv, Xuying Yang, Xianghong Liu

**Affiliations:** Department of Emergency Medicine, Ganzhou People’s Hospital, Ganzhou, 341000, China; Department of Scientific Affairs, Hugobiotech Co., Ltd., Beijing 100176, China; Department of Neurology, Ganzhou People’s Hospital, Ganzhou, 341000, China

**Keywords:** metagenomic next-generation sequencing, infection after inguinal hernia repair, anaerobes, mixed infection, adjustment of the antibiotic regimen, case report

## Abstract

Infection following inguinal hernia repair (IHR) is uncommon. Rational use of antibiotics can significantly improve the prognosis of patients. However, accurately identifying the pathogen involved is usually challenging. This case report describes a patient who developed intermittent fever after undergoing open preperitoneal tension-free repair of a bilateral inguinal hernia. The scrotal fluid specimen was cultured and subjected to metagenomic next-generation sequencing (mNGS). Culture revealed the presence of *Enterococcus faecalis* (a facultative anaerobe). However, mNGS detected *E. faecalis* along with multiple anaerobic bacteria including *Bacteroides thetaiotaomicron*, *Parabacteroides distasonis*, and *Levyella massiliensis*. The patient was finally diagnosed with a mixed infection of *E. faecalis* and multiple anaerobes, and his condition was effectively controlled after timely adjustment of the antibiotic regimen. Treating postoperative infections with multiple concurrent conditions can be challenging. mNGS is valuable for the accurate diagnosis and treatment of infections, as it not only can further verify the culture results, but also assist clinicians in ruling out pulmonary infection caused by hematogenous dissemination after IHR in patients.

## 1 Introduction

Inguinal hernia is a common surgical condition, and its repair is the most common general surgical procedure, with studies showing an annual surgical rate of approximately 0.2% in the United States [1]. Inguinal hernia repair (IHR) surgery can be performed using both suture-based and mesh-based techniques and can be open or minimally invasive [2]. Currently, the vast majority of IHR repair tissue procedures are performed by placing a tension-free synthetic mesh [3]. Studies have shown that the postoperative infection rate in IHR ranges from 2.4 to 4.9% [4,5]. IHR infections are usually caused by *Staphylococcus* spp., *Enterococcus* spp., and gram-negative bacteria. Although infection is infrequent, it is very severe once it occurs [6]. *Staphylococcus* spp. and gram-negative bacteria are always considered in clinical empirical medication [7]. Timely and accurate diagnosis of infectious pathogens becomes crucial for a successful postoperative recovery.

In this case, a patient who underwent an open preperitoneal tension-free repair of a bilateral inguinal hernia presented with persistent, intermittent fever 5 days after surgery with negative scrotal effusion culture. Seven days later, scrotal effusion was obtained again and sent for culture, meanwhile metagenomic next-generation sequencing (mNGS) was performed 2 days later, both of which detected *Enterococcus faecalis*, while the mNGS also detected *Bacteroides thetaiotaomicron*, *Parabacteroides distasonis*, and *Levyella massiliensis*. The antibiotic regimen was adjusted accordingly, and the patient’s condition improved.

## 2 Case presentation

On November 20, 2019 (10 days before admission to our hospital), a 72-year-old male patient was admitted to a local hospital for bilateral inguinal hernias and underwent open tension-free repair surgery without complications. The patient had a previous history of cerebral infarction with left limb dysfunction. Five days after the surgery, the patient developed left-sided chest pain with fever, cough, and copious white sputum. Chest computed tomography (CT) indicated bilateral lung infection and pleural effusions. Despite treatment with anti-infective and antiasthmatic medications, the chest pain persisted.

**Figure 1:**
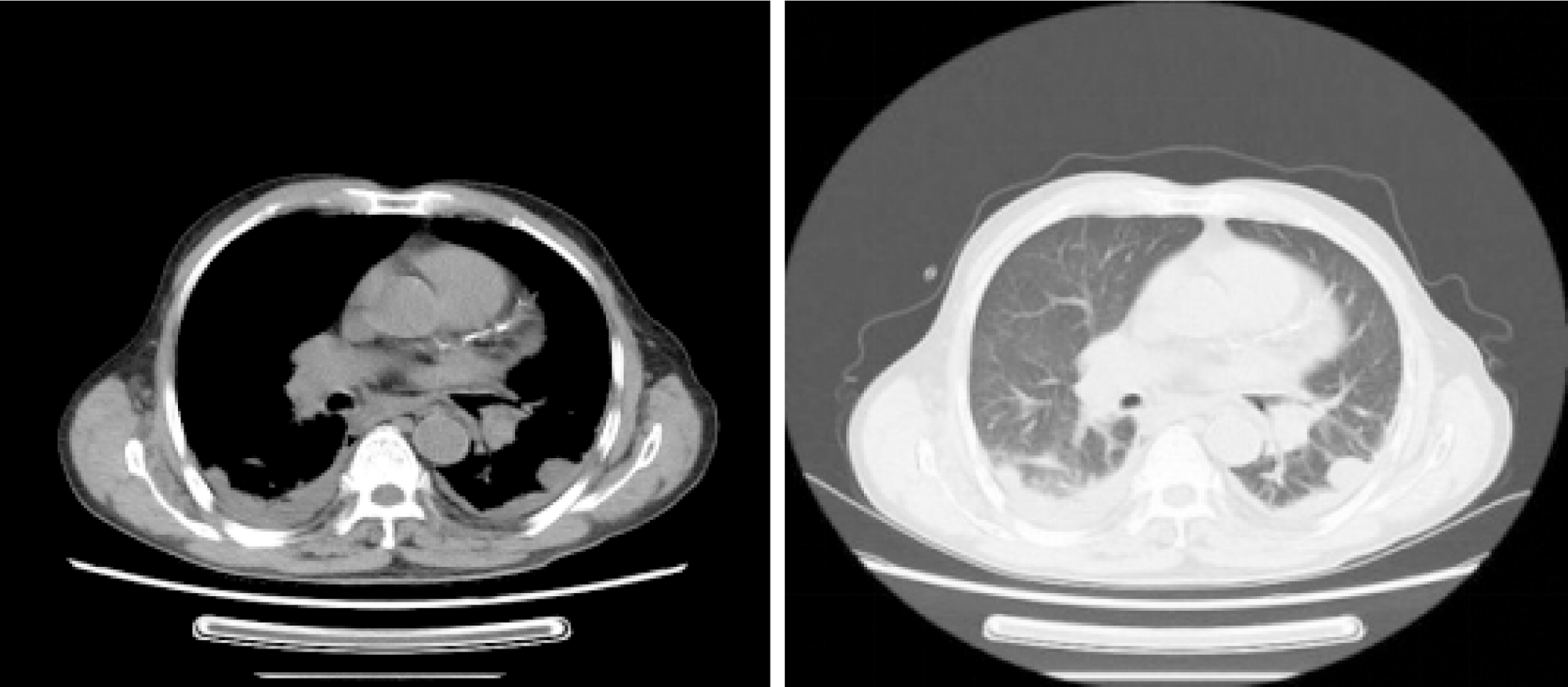
CT scan of the patient chest.

On December 1, 2019, the patient was transferred to our hospital for further treatment. Physical examination showed normal vital signs, clear mind, and normal thorax. Bilateral breath sounds were coarse with a small amount of moist rales. The patient had surgical incisions (5 cm) in the bilateral inguinal regions, which appeared swollen, red, and painful. Re-examination of chest and abdominal CT (Figure 1) revealed bilateral lung infections, bilateral pleural effusions, abnormal shadows in bilateral scrotum and inguinal regions, calcification in the aortic wall and coronary wall, multiple stones in the right kidney, and suspected hyperplasia in the left adrenal gland.

The blood gas analysis revealed a pH of 7.51 and a partial pressure of oxygen of 43 mmHg. The procalcitonin (PCT) level was 0.87 ng/mL (normal range: 0–0.5 ng/mL), indicating an elevated value. The C-reactive protein level was 237.9 mg/L (normal range: 0–6.0 mg/L), indicating a significantly elevated value. The blood routine examination indicated a high white blood cell (WBC) count of 21.88 × 10^9^/L (normal range: 3.5–9.5 × 10^9^/L), with neutrophils accounting for 90.50% (normal range: 40–75%). Additionally, the patient had low levels of red blood cells (3.53 × 10^12^/L) and hemoglobin (111 g/L). The liver function, electrolytes, coagulation function, and other biochemical examinations showed mild abnormalities. Based on these findings, the initial clinical diagnosis was pulmonary infection with pulmonary embolism, type I respiratory failure, and infection after bilateral IHR.

On the following day after admission (December 2), the patient underwent a color Doppler ultrasonography, which suggested scrotal effusion, but an abscess could not be ruled out. Culture using scrotal effusion (yellowish) after puncture was negative. Empirical antibiotic therapy conducted with piperacillin sodium and tazobactam sodium (4.5/0.5 g equivalent every 8 h by intravenous drip) in combination with linezolid (600 mg every 8 h by intravenous drip) were given. However, the condition continued to worsen.

On Day 5 after admission (December 5), color Doppler ultrasonography re-examination revealed scrotal and inguinal edema, which might be caused by large omentum and hernia contents. Smear using scrotal fluid after another puncture was still negative. During this period, the patient still had fever, WBC (18.07 × 10^9^/L) and PCT (0.59 ng/mL) were lower than before. Scrotal swelling diminished in size, especially on the right side. The antibiotic regimen continued.

**Figure 2:**
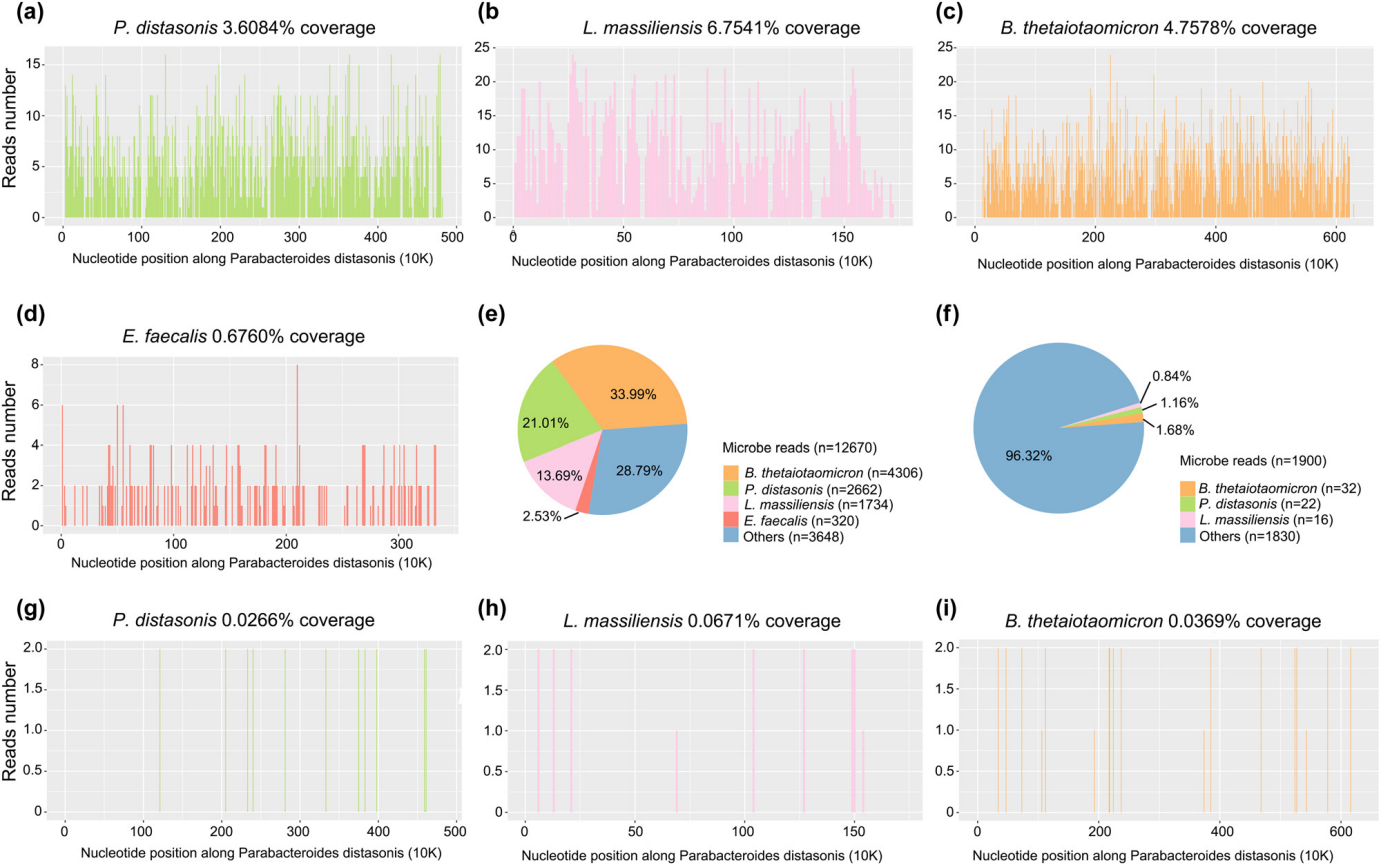
Results of mNGS using scrotal fluid and blood. (a–d) The genome coverage of detected *P. distasonis*, *L. massiliensis*, *B. thetaiotaomicron*, and *E. faecalis* by mNGS using scrotal fluid was 3.61, 6.75, 4.76, and 0.68%, respectively. (e and f) The detected specific reads number and the percentage of each pathogen by mNGS using scrotal fluid and blood in this patient. (g and h) The genome coverage of detected *P. distasonis*, *L. massiliensis*, and *B. thetaiotaomicron* by mNGS using blood was 0.07, 0.04 and 0.03%, respectively.

**Figure 3:**
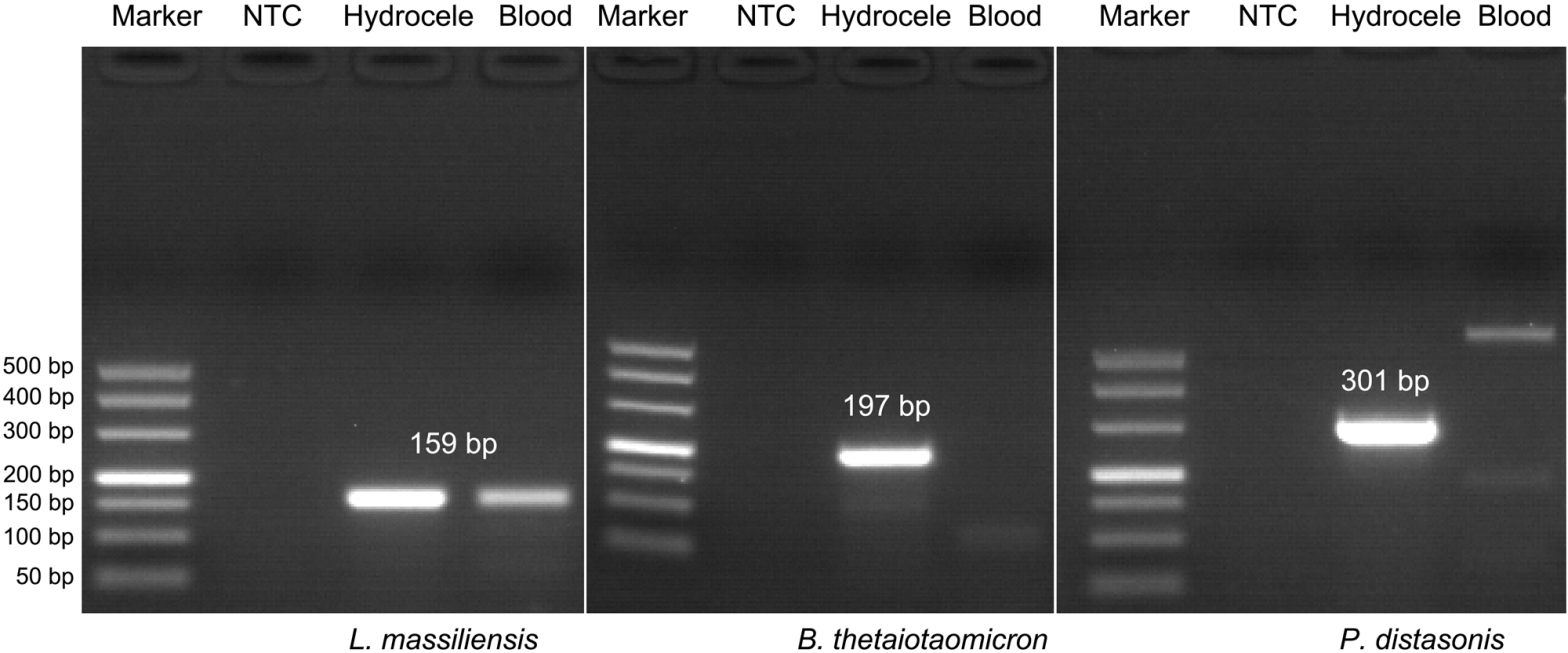
PCR results of *L. massiliensis*, *B. thetaiotaomicron*, and *P. distasonis* using hydrocele and blood in this patient. *L. massiliensis* was positive in both hydrocele and blood. *B. thetaiotaomicron* and *P. distasonis* were positive in hydrocele but negative in blood. The PCR amplification primers (target 16S) for *B. thetaiotaomicron* were 5ʹ-GCAAACTGGAGATGGCGA-3ʹ (Forward) and 5ʹ-AAGGTTTGGTGAGCCGTTA-3ʹ (Reverse) with amplified fragment of 197 bp. The PCR amplification primers (target 16S) for *P. distasonis* were 5ʹ-AATACCGCATGAAGCAGG-3ʹ (Forward) and 5′-GACACGTCCCGCACTTTA-3′ (Reverse) with amplified fragment of 301 bp. The PCR amplification primers (target 16S) for *L. massiliensis* were 5′-TAGGTGTCGGGTGTCAAAGC-3′ (Forward) and 5′-CCTGGTAAGGTTCTTCGCGT-3′ (Reverse) with amplified fragment of 159 bp. PCR amplification conditions were 94℃, 5 min; 40 cycles (94℃, 30 s; 53℃, 30 s; 72℃, 30 s); 72℃, 5 min. NTC in the image refers to no template control.

**Figure 4:**
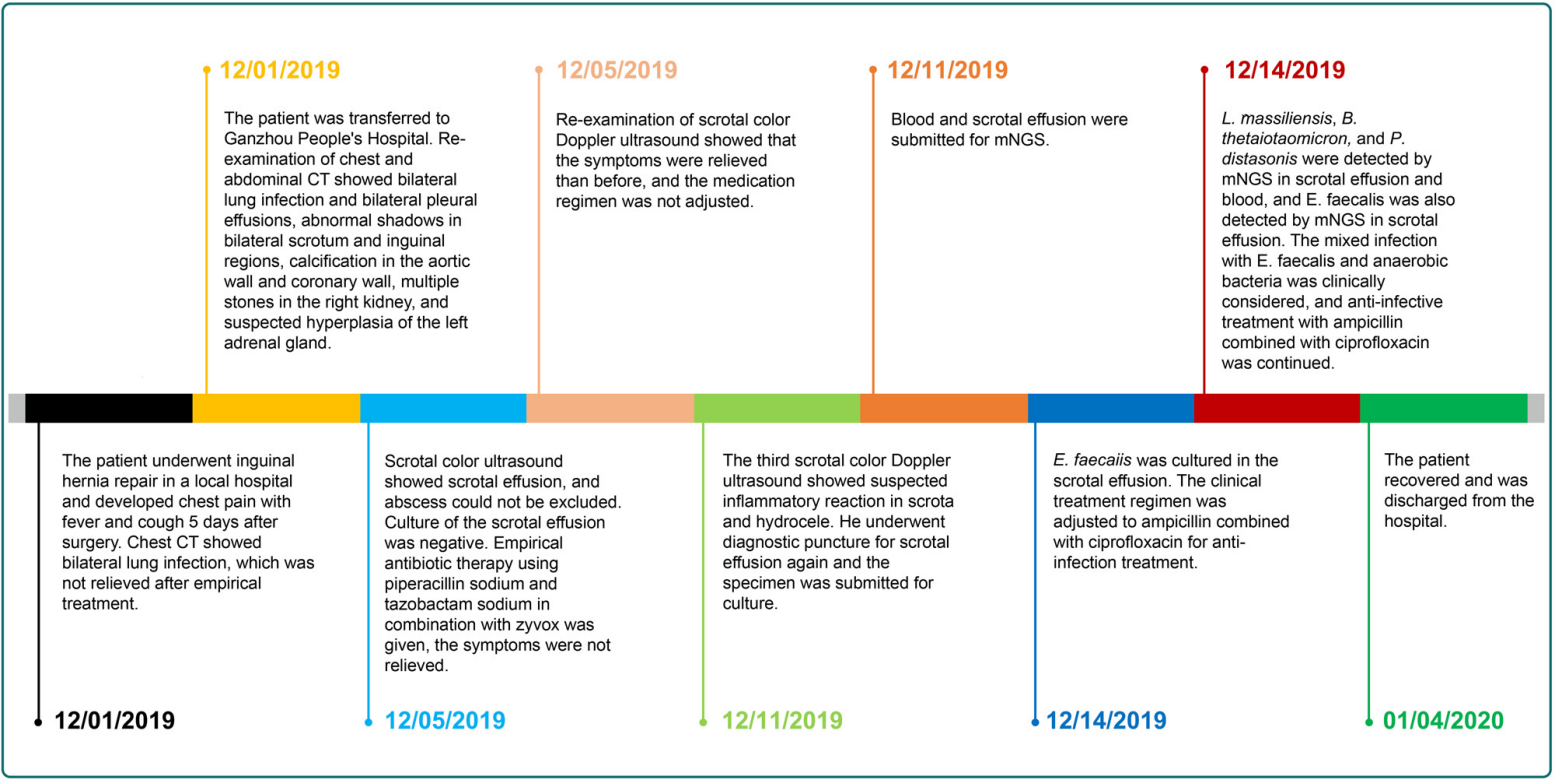
Progression diagram of the patient’s condition.


On Day 9 (December 9), the third scrotal color Doppler ultrasonography revealed mixed echogenic masses in the scrotum bilaterally, inflammatory masses could not be excluded, and mild syringomyelia bilaterally. The patient underwent puncture of scrotal effusion. The fluid was soy sauce colored with a foul odor. Culture was performed using Columbia Blood Agar and China Blue Agar at 37℃ for 5 × 24 h, indicating *E. faecalis* (December 12). The results of the antibiotic susceptibility tests indicated that ampicillin, gentamicin, teicoplanin, rifampicin, vancomycin, ciprofloxacin, furantoin, and linezolid are effective against the isolates, but tetracycline showed resistance. Meanwhile, blood and scrotal effusion were sent for mNGS (Hugobiotech, Beijing, China) on December 11 (Day 11), both of which revealed *L. massiliensis*, *B. thetaiotaomicron*, *P. distasonis*, and *E. faecalis* (Figure 2a–i). Polymerase chain reaction (PCR) using scrotal effusion confirmed the mNGS detection, while blood PCR only detected *L. massiliensis* (Figure 3). The detailed mNGS process can be found in Yu et al. [8]. Accordingly, the treatment regimen was adjusted to ampicillin (3 g every 6 h by intravenous drip) combined with ciprofloxacin (0.4 g every 12 h by intravenous drip). The patient’s symptoms gradually improved (Figure 4). He was discharged on January 14, 2020).

**Consent to publish**: Written informed consent was obtained from the patient for publication of this case report and any accompanying images. A copy of the written consent is available for review by the Editor of this journal upon request.
**Statement of informed consent obtained:** Informed written consent has been obtained from the patient and can be provided at any time upon request.

## 3 Discussion and conclusions

IHR surgery carries associated postoperative risks, including prolonged pain, bleeding, nerve injury, peripheral vascular injury, and damage to adjacent organs, and most urgently, resolution of postoperative infection. Clinically, pathogens should be identified by culture in the diagnosis of postoperative infection. However, because the results of the culture method are often false negative, doctors sometimes have to consider empirical medication. Culture method is the gold standard testing for pathogen identification, but it is time-consuming and misses some pathogens, such as anaerobes [9,10]. Current culture techniques in the clinic have low detection efficacy for anaerobes [11]. Postoperative infections are often acute and rapidly progressive, and culture is clearly not the most appropriate method of diagnosis.

In this case, the patient was discharged after adjusting the antibiotic regimen according to the mNGS results (from piperacillin sodium and tazobactam sodium in combination with linezolid to ampicillin combined with ciprofloxacin). The results illustrate that pathogens in this patient were rare and culture was limited for clinical identification of anaerobes. As an unbiased, culture-free method compared to culture, mNGS identifies a comprehensive range of pathogens, including rare ones, and provide a full view of a patient’s infection. The patient’s specimen was not sent for mNGS at the same time as the first scrotal effusion culture, which might have caused some delay in the precise medication of postoperative infection.

Pathogens were detected in scrotal effusion and blood by culture, PCR, and especially mNGS detection. The clinicians took into account the patient’s clinical features in combination with various clinical factors to exclude pulmonary infection caused by hematogenous dissemination. Although early empirical treatment did not bring clinical benefits for postoperative infection, piperacillin sodium and tazobactam sodium combined with linezolid for anti-infection also played a certain role in controlling pulmonary infection. Subsequently, a mixed postoperative infection with *E. faecalis* and anaerobes was clinically judged based on the foul odor of the scrotal fluid drainage. *B. thetaiotaomicron*, *P. distasonis*, *L. massiliensis*, and *E. faecalis* are important bacteria in human gut. Consider the previous IHR of this patient, the origin of these causative bacteria might be from gut.

The coexistence of multiple conditions makes the diagnosis and treatment of postoperative infection for the patient quite difficult. mNGS is important for the accurate diagnosis and treatment of diseases. By timely adjustment of the antibiotic regimen and puncture drainage, the patient’s condition was effectively controlled and was discharged from the hospital eventually.

## Abbreviations

CT: computed tomography
CTA: CT angiography
IHR: inguinal hernia repair
mNGS: metagenomic next-generation sequencing
PCR: polymerase chain reaction
PCT: procalcitonin
WBC: white blood cell
